# Repeated Parallel Evolution of Parental Care Strategies within *Xenotilapia*, a Genus of Cichlid Fishes from Lake Tanganyika

**DOI:** 10.1371/journal.pone.0031236

**Published:** 2012-02-08

**Authors:** Michael R. Kidd, Nina Duftner, Stephan Koblmüller, Christian Sturmbauer, Hans A. Hofmann

**Affiliations:** 1 The University of Texas at Austin, Section of Integrative Biology, Institute for Neuroscience, Austin, Texas, United States of America; 2 Texas A&M International University, Department of Biology & Chemistry, Laredo, Texas, United States of America; 3 University of Graz, Department of Zoology, Graz, Austria; 4 The University of Texas at Austin, Institute for Cellular and Molecular Biology, Austin, Texas, United States of America; Biodiversity Insitute of Ontario - University of Guelph, Canada

## Abstract

The factors promoting the evolution of parental care strategies have been extensively studied in experiment and theory. However, most attempts to examine parental care in an evolutionary context have evaluated broad taxonomic categories. The explosive and recent diversifications of East African cichlid fishes offer exceptional opportunities to study the evolution of various life history traits based on species-level phylogenies. The *Xenotilapia* lineage within the endemic Lake Tanganyika cichlid tribe Ectodini comprises species that display either biparental or maternal only brood care and hence offers a unique opportunity to study the evolution of distinct parental care strategies in a phylogenetic framework. In order to reconstruct the evolutionary relationships among 16 species of this lineage we scored 2,478 Amplified Fragment Length Polymorphisms (AFLPs) across the genome. We find that the Ectodini genus *Enantiopus* is embedded within the genus *Xenotilapi*a and that during 2.5 to 3 million years of evolution within the *Xenotilapia* clade there have been 3–5 transitions from maternal only to biparental care. While most previous models suggest that uniparental care (maternal or paternal) arose from biparental care, we conclude from our species-level analysis that the evolution of parental care strategies is not only remarkably fast, but much more labile than previously expected.

## Introduction

Cost-benefit analysis has generated important insights into the evolution of parental care (reviewed by [1]), modeling factors such as reproductive effort [2], [3], assurance of paternity [4], mate guarding [5], predation risk [6], [7], and the opportunity for additional matings [8]–[10]. For species that utilize external fertilization, a “stepping stone” model has been proposed [11], [12] in which the ancestral “no care state” is followed by paternal only care (initiated by a need to assure paternity, or as an extension of territoriality), transitioning to biparental care (initiated by an increased need to provide for or defend the offspring), finally resulting in maternal only care (initiated by male desertion). The “stepping stone model” has been broadly applied to fishes and amphibians and has been used to explain the unusually high proportion of maternal mouthbrooding species among cichlid fishes [13], [14]. Nevertheless, recent phylogenetic analyses of parental care evolution in fish [12] and frogs [15] have questioned the extent to which biparental care is an intermediate stage between paternal only and maternal only care.

All known species of fish from the family Cichlidae perform extended parental care and exhibit a wide range of parental care strategies, including maternal only, paternal only, biparental, alloparental, and even communal/cooperative parental care [13], [16], [17]. It has been suggested that biparental substrate guarding is the ancestral parental care state for the family Cichlidae due to its ubiquitous geographic distribution and the presence of specialized egg morphology that would otherwise have to have evolved repeatedly [13], [18], [19]. Many substrate guarding species move eggs or larvae in their mouths from one location to another within their territory [13], [20], which is thought to have been the evolutionary antecedent to biparental mouthbrooding [13], [19], [21], [22]. The “stepping stone” model suggests that the transition to maternal only mouthbrooding is the result of male desertion [8]. This hypothesis is supported by observations of the tilapiine cichlid species *Sarotherodon galilaeus* [23] and *Sarotherodon caroli* (pers. obs. Kidd), which display an initial pair-bond before spawning that dissolves after spawning is complete.

The hypothesis that biparental mouthbrooding is the intermediate parental care state between biparental substrate spawning and maternal only mouthbrooding has received support from both cost/benefit modeling [9] and phylogenetic analyses [24], [25]. Game theory modeling, based on empirical measurements of costs and benefits of care in the behaviorally plastic tilapiine species *Sarotherodon galilaeus*, suggest that biparental care is an evolutionarily stable strategy only when the operational sex ratio is heavily male biased and when clutch size is larger than either sex can incubate alone [9]. In any other circumstance, uniparental care (either male or female) will be the optimal strategy. Previous phylogenetic analyses of parental care strategies in the family Cichlidae have identified many transitions from biparental mouthbrooding to maternal only mouthbrooding and only a few possible transitions from maternal only care to biparental care [24], [25]. As suggested by Gonzalez-Voyer *et al*. [26], phylogenetic analyses at higher taxonomic levels lack the power to fully account for the substantial variation in parental care strategies within families of fish that are only revealed when examining species level phylogenies.

The 13–17 species in the genus *Xenotilapia*, part of the endemic Lake Tanganyika tribe Ectodini, are all mouthbrooders that exhibit either monogamous-biparental or polygamous maternal only care of offspring [27]–[29] and utilize a wide variety of habitats [27], [30]. Based on the morphology of the pharyngeal apophysis, the genera *Asprotilapia*, *Enantiopus* and *Microdontochromis* were separated from *Xenotilapia* [27], [31], [32], however the validity of these genera has been questioned [33]. The natural diversity of parental care strategies exhibited by this clade provides a tremendous opportunity to examine the molecular and neural basis of social behavior and brain evolution in a powerful comparative context [34], [35]. Unfortunately, the recent and rapid radiation of this group, within the last 2.5–3 million years [28], has made phylogenetic analyses of the clade a challenge.

**Figure 1:**
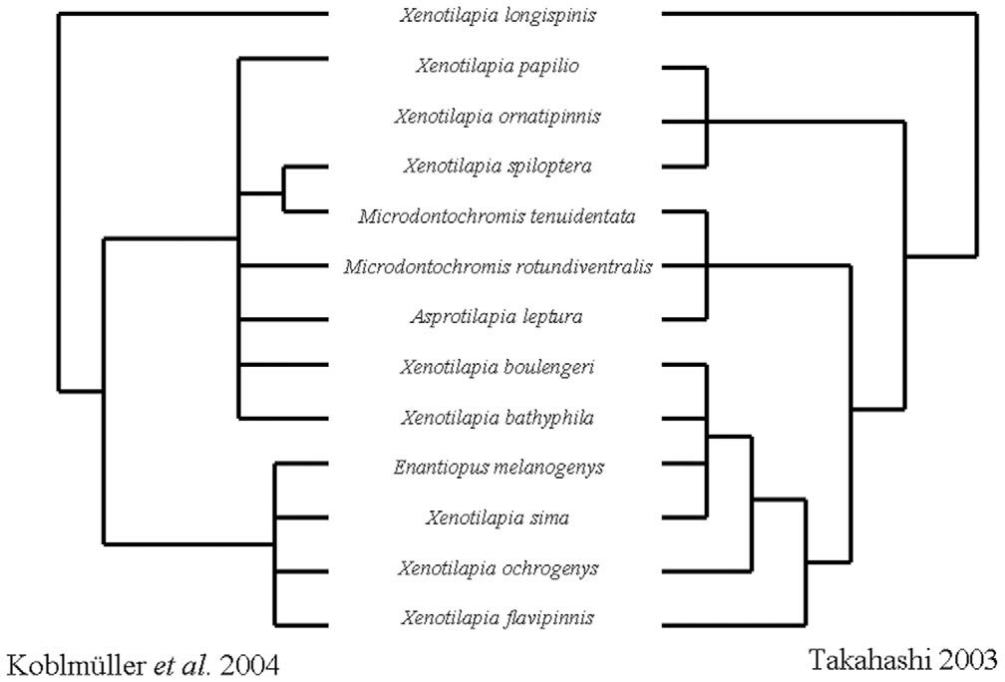
Comparison of contrasting recent phylogenetic hypotheses of the relationships between species of the *Xenotilapia* lineage redrawn from Koblmüller *et al*. [**28**] and Takahashi [**33**].

While the monophyly of the Ectodini lineage is supported by anatomy [27], [31], [32] and sequence data [36]–[39], the evolutionary relationships between genera within the Ectodini remain unclear. Two recent phylogenetic analyses of this clade (Fig. 1) agree that the genera *Xenotilapia*, *Microdontochromis*, *Enantiopus*, and *Asprotilapia* form a distinct clade within Ectodini and that the genus *Xenotilapia* is paraphyletic with respect to the other genera [28], [33]. Unfortunately, neither phylogenetic analysis is adequate to reconstruct the evolution of parental care strategies within this lineage. Takahashi's [33] cladistic analysis of 14 morphological characters was unable to provide enough resolution to determine the relationships between many of the species within the clade. Koblmüller *et al*. [28] were able to identify at least two transitions between parental care states and that biparental care evolved from maternal only care at least once. However, reconstructing the evolutionary relationships between species within a rapidly radiating clade is often confounded by the retention of ancestral polymorphisms [40]–[42] or hybridization, especially if phylogenetic inference is based on a single gene or linked loci [43], [44]. Techniques that survey thousands of independent nuclear loci, such as Amplified Fragment Length Polymorphisms (AFLP), overcome these challenges and have emerged as the primary tool for elucidating the relationships between recently and rapidly evolved cichlid species [43]–[51]. In the present study we use AFLP, a genomic fingerprinting technique [52], [53], to examine the evolution of parental care within the *Xenotilapia* clade. Since biparental care is generally associated with monogamous mating systems and maternal only care with polygamous mating systems [16], our phylogenetic analysis provides the comparative context necessary to elucidate the proximate mechanisms underlying the evolution of parental care and alternative mating strategies.

## Materials and Methods

### Collection of Samples

We sampled 32 individuals from 11 species within the *Xenotilapia* clade (1–5 individuals each). Also included were one individual each from the Ectodini species *Callochromis macrops*, *C. stappersii*, *Cyathopharynx furcifer*, *Ophthalmotilapia nasuta*, and *O. ventralis* as outgroups. Samples were collected during several expeditions to Lake Tanganyika, or acquired from the aquarium trade (Table 1). Data on parental care type came from the literature [14], [16], [27], [29], [54]–[56] and were confirmed by observations of parental care behavior in both field and laboratory for *Xenotilapia ornatipinnis*, *X. flavipinnis*, *X.* sp. “papilio sunflower”, *X. spiloptera*, *X. ochrogenys*, *Microdontochromis tenuidentata*, *Enantiopus melanogenys*, *Asprotilapia leptura*.

**Table 1:** Taxa of cichlids sampled for AFLP fingerprinting analysis.

Species	Collection Site	Parental Care Strategy
*Asprotilapia leptura*	Isanga	Biparental Mouthbrooder
*Asprotilapia leptura*	Isanga	Biparental Mouthbrooder
*Asprotilapia leptura*	Tongwa	Biparental Mouthbrooder
*Callochromis macrops*	Ndole	Maternal Mouthbrooder
*Callochromis stappersii*	Aquarium Trade	Maternal Mouthbrooder
*Cyathopharynx furcifer*	Toby's lodge	Maternal Mouthbrooder
*Enantiopus melanogenys*	Aquarium Trade	Maternal Mouthbrooder
*Enantiopus melanogenys*	Aquarium Trade	Maternal Mouthbrooder
*Enantiopus melanogenys*	Aquarium Trade	Maternal Mouthbrooder
*Microdontochromis tenuidentata*	Mpulungu	Maternal Mouthbrooder
*Microdontochromis tenuidentata*	Mpulungu	Maternal Mouthbrooder
*Microdontochromis tenuidentata*	Mpulungu	Maternal Mouthbrooder
*Ophthalmotilapia nasuta*	Nakaku	Maternal Mouthbrooder
*Ophthalmotilapia ventralis*	Wonzye	Maternal Mouthbrooder
*Xenotilapia bathyphila*	Mbita Island West	Maternal Mouthbrooder
*Xenotilapia boulengeri*	Chimba	Biparental Mouthbrooder
*Xenotilapia boulengeri*	Kalambo Lodge	Biparental Mouthbrooder
*Xenotilapia flavipinnis*	Kantalamba	Biparental Mouthbrooder
*Xenotilapia flavipinnis*	Aquarium Trade	Biparental Mouthbrooder
*Xenotilapia flavipinnis*	Katete	Biparental Mouthbrooder
*Xenotilapia flavipinnis*	Kigoma	Biparental Mouthbrooder
*Xenotilapia ochrogenys*	Kavalla, Congo	Maternal Mouthbrooder
*Xenotilapia ochrogenys*	Kavalla, Congo	Maternal Mouthbrooder
*Xenotilapia ochrogenys*	Kavalla, Congo	Maternal Mouthbrooder
*Xenotilapia ornatipinnis*	Aquarium Trade	Maternal Mouthbrooder
*Xenotilapia ornatipinnis*	Aquarium Trade	Maternal Mouthbrooder
*Xenotilapia sima*	Aquarium Trade	Maternal Mouthbrooder
*Xenotilapia sima*	Aquarium Trade	Maternal Mouthbrooder
*Xenotilapia* sp. “papilio sunflower”	Chituta Bay	Biparental Mouthbrooder
*Xenotilapia* sp. “papilio sunflower”	Chituta Bay	Biparental Mouthbrooder
*Xenotilapia* sp. “papilio sunflower”	Aquarium Trade	Biparental Mouthbrooder
*Xenotilapia* sp. “papilio sunflower”	Aquarium Trade	Biparental Mouthbrooder
*Xenotilapia spiloptera*	Kapembwa	Biparental Mouthbrooder
*Xenotilapia spiloptera*	Chimba	Biparental Mouthbrooder
*Xenotilapia spiloptera*	Mbita Island East	Biparental Mouthbrooder
*Xenotilapia spiloptera*	Kigoma	Biparental Mouthbrooder
*Xenotilapia spiloptera*	Kigoma	Biparental Mouthbrooder

### Ethics Statement

All work was performed in compliance with the Institutional Animal Care and Use Committee at The University of Texas at Austin (#06072402) and Harvard University (#22–22). Research permits (#2003-192-ER-98-52) for field observations and sample collection were issued by the Tanzania Commission for Science and Technology (COSTECH).

### AFLP analysis

Genomic DNA was extracted from either the pectoral or caudal fin tissue using a standard phenol-chloroform protocol [57]. Efficiency of the extraction process was quantified using a Nanodrop ND-1000. Restriction-ligation and PCR protocols followed Kidd *et al*. [47], with the exception that the selective amplification utilized 12 different primer pair combination with two nucleotide extensions (E-ACA, M-CAA, M-CAG; E-ACC, M-CAA, M-CAT, M-CTA; E-ACT, M-CAA, M-CAC, M-CAT, M-CTA; E-AGC, M-CAA; E-AGG, M-CTG, M-CTT). Fragments were separated using a Beckman Coulter CEQ 8000 capillary sequencer. Peaks were scored using a quartic model with a slope threshold of 2.0% and relative peak height of 5.0% [47]. Bands were scored as present/absent using Beckman Coulter's Fragment Analysis Module, however, since automated scoring can be unreliable [58], [59], the presence of each fragment was confirmed manually. Fragments between 90–500 bp in size were binned (1 nucleotide bin width) using Beckman Coulter's AFLP Analysis Software. The binary output was imported to an Excel spreadsheet and formatted for PAUP v. 4.0b8 [60].

### Phylogenetic analyses

A matrix of genetic distances was generated using Nei & Li's Distance [61], which was used to generate a phylogram constructed from 10,000 bootstrap replicates using a neighbor joining algorithm in PAUP v.4.0b8 [60]. The data were tested for hierarchical structure by analyzing the frequency and distribution of tree lengths for 1,000,000 randomly generated trees [62]. An additional phylogram was constructed using maximum parsimony by implementing PAUP's default settings for a full heuristic search with 10,000 bootstrap replicates. We evaluated the effects of reticulation on the structure of this phylogeny using the homoplasy excess test [43], [63] following Kidd *et al*. [47]. We tested for the parallel evolution of parental care strategies by designing constraint trees that assumed a monophyletic origin for each parental care state. Using the same parameters described above, PAUP identified the best tree that included the constraint and Shimodaira-Hasegawa tests (SH) were used to compare the alternate topological hypotheses [64]. We imported current parental care strategies into MESQUITE v.2.0 [65], in order to perform a parsimony reconstruction of the ancestral parental care states using unordered character states, which allows equal probability of transition between bi-parental and maternal only care and a maximum likelihood reconstruction using a Markov k-state one parameter model.

## Results

**Figure 2:**
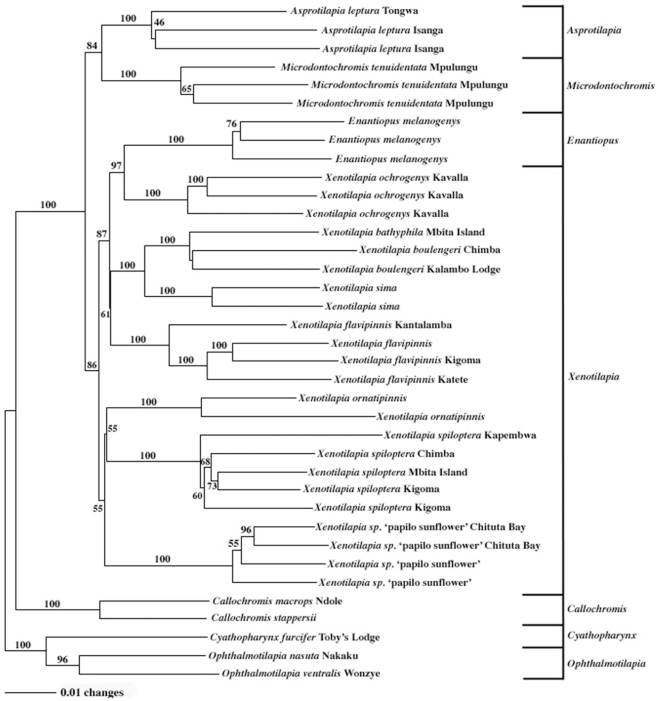
Neighbor joining dendrogram of the *Xenotilapia* lineage based on Nei & Li's genetics distance calculated from 2,478 AFLP loci. Numbers at each node indicate bootstrap values (from 10,000 replicates) for that node. Lines on the right indicate current generic assignment of each taxon. The tree was rooted with *Opthalmotilapia nasuta* and *O. ventralis*.

Twelve primer pair combinations generated 3,588 characters (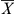 = 299.0 per primer pair). Of these, 2,478 were polymorphic and informative (57.9 to 75.9% per primer pair). A plot of the length of 1,000,000 random trees demonstrated significant non-random structure to the data set (g_1_ = −0.68395, 37 samples, p<0.01). These data were used to construct a distance tree (Fig. 2) with a mean bootstrap value of 86.0%. All but two nodes were resolved above 50% and 25 nodes were resolved above 75%. With the exception of *Xenotilapia boulengeri*, all species form monophyletic clusters (supported by 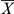 = 94.2% bootstrap support). Parsimony methods yielded a single tree that was topologically identical to the distance tree (SH test, *p* = 0.388), but differed in bootstrap support for specific nodes (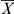 = 77.0% bootstrap support overall). Although Seehausen's [63] homoplasy excess test has been shown to be very sensitive to the effects of hybridization on a phylogeny [43], [47], [49], [50], our analysis failed to identify any instances of reticulation within this data set.

After rooting the tree using *Opthalmotilapia nasuta* and *O. ventralis*, the phylogeny recovers the expected relationships between nested outgroups with *Cyathopharynx furcifer* as sister to the *Ophalmotilapia* clade and *Callochromis macrops* and *C. stappersii* as sister to the *Xenotilapia* clade. *Asprotilapia leptura* and *Microdontochromis tenuidentata* form a reciprocally monophyletic clade, sister to rest of the *Xenotilapia* species. The species pair *Enantiopus melanogenys* and *Xenotilapia ochrogenys* cluster with a large assemblage consisting of *X. bathyphila*, *X. boulengeri*, *X. sima*, and *X. flavipinnis*. This group is sister to a less resolved lineage that includes *X. ornatipinnis*, *X. spiloptera*, and *X.* sp. “papilio sunflower”. The topology of this phylogram was significantly different (SH test, p<0.0001) from the topology generated by Koblmüller *et al*. [28] from mtDNA sequence data. However, our tree was topologically indistinguishable (SH test, p = 0.7161) from Takahashi's [33] consensus tree (Fig. 1).

**Figure 3:**
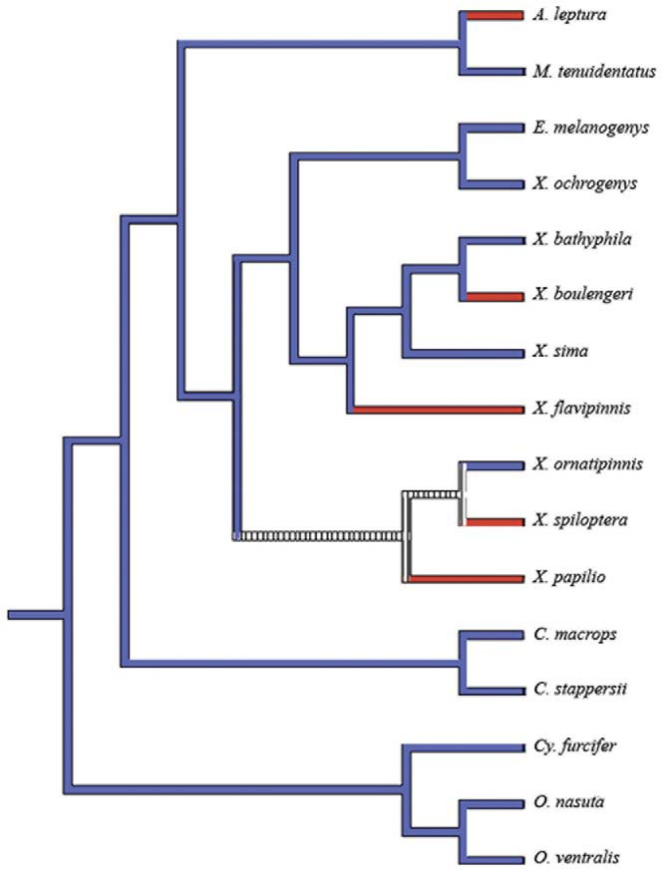
Convergent evolution of mating strategies within the Ectodini/*Xenotilapia* clade from Lake Tanganyika. Ancestral character state reconstruction by maximum parsimony revealed multiple transitions from biparental (red) to maternal only care (blue), which would require the repeated evolution neural and endocrine pathways regulating parental care and mate choice decisions. Our analysis was unable to resolve the parental care state for the ancestor of the clade consisting of *X. ornatipinnis*, *X. spiloptera* and *X*. sp. “papilio sunflower” (barred).

Neither maternal only nor biparental care character states define a monophyletic lineage (SH test, p<0.0001 for both conditions). While our maximum likelihood analysis was unable to reconstruct the ancestral parental care states, our maximum parsimony analysis suggests that, when accounting for topological uncertainty as indicated by poorly supported nodes, maternal only mouthbrooding is the ancestral state for the *Xenotilapia* lineage and that there have been 3–5 transitions from maternal only to biparental mouthbrooding (Fig. 3).

## Discussion

### Evolution and taxonomic status of the Genus Xenotilapia

Our results add to the growing evidence that the genus *Xenotilapia* is paraphyletic and in need of revision [28], [33]. Greenwood [31], Poll [27] and Takahashi *et al*. [32] used the shared “*Tropheus*-type” pharyngeal apophysis to separate the genera *Enantiopus*, *Asprotilapia*, and *Microdontochromis* from *Xenotilapia*, which possesses a “*Haplochromis*-type” pharyngeal apophysis. However in a recent reexamination of the lineage, Takahashi [33] found that *X. caudafasciata*, *X. papilio*, and *X. spiloptera* also share the “*Tropheus*-type” morphology and suggested that this trait was inappropriate for splitting the genera. In this phylogeny, *Enantiopus melanogenys* clusters with *Xenotilapia ochrogenys* and is clearly embedded within the *Xenotilapia* lineage (Fig. 2). Both Koblmüller *et al*. [28] and Takahashi [33] suggest that these two species share a common clade within the *Xenotilapia* lineage, although neither had sufficient resolution to determine their evolutionary relationship in finer detail.

Our results do not indicate that the genus *Xenotilapia* is paraphyletic with respect to *Asprotilapia leptura* and *Microdontochromis tenuidentata*, which form a reciprocally monophyletic lineage sister to the other *Xenotilapia* species examined here, suggesting that placement of these species into separate genera by Greenwood [31] and Poll [27] was valid. However, we did not survey samples of *Xenotilapia longispinis*, which Takahashi [33] and Koblmüller *et al*. 's [28] analyses suggest is basal to all of the *Xenotilapia* taxa, including *Asprotilapia leptura*, *Microdontochromis tenuidentata* and *M. rotundiventralis*. Considering the position of *X. longispinis* in these other analyses and the topological congruence between our tree and that of Takahashi [33], the genus *Xenotilapia* is likely paraphyletic with respect to *Asprotilapia* and *Microdontochromis* as well as the genus *Enantiopus*.

### Evolution of parental care strategies

Mapping parental care states onto our phylogenetic hypothesis suggests that maternal only mouthbrooding within a polygamous mating system was the ancestral parental care state for the *Xenotilapia* lineage, which was followed by multiple independent transitions to biparental care and monogamy (Fig. 3). Koblmüller *et al*. [28] suggested that there have been multiple transitions from maternal only to biparental care, but their analysis lacked the resolution to reject the alternative possibility, that the ancestral *Xenotilapia* was a biparental mouthbrooder and that there had been multiple transitions to maternal only care. Six species from the *Xenotilapia* lineage were not represented in this analysis, which include three species that exhibit maternal only care (*X. burtoni*, *X. nigrolabiata*, *M. rotundiventalis*) and three species for which there is currently no, or conflicting information available concerning their parental care strategies (*X. nasus*, *X. caudafasciata*, *X. longispinis*). The limited resolution of previous phylogenies for this group [28], [33], the fact that two species (*X. burtoni, X. nasus*) have not been examined in any phylogenetic analysis, and the incomplete information concerning the parental care strategies for some species, all indicate that further studies will be necessary to fully elucidate the number of transitions between parental care strategies during the diversification of this clade. However, our results suggest that the evolution of parental care strategies may be more labile then previously recognized, supporting recent findings in fishes [12] and frogs [15] and suggesting that the view of biparental care as simply an intermediate step may be overly simplistic.

Transitions to biparental mouthbrooding from female only mouthbrooding are expected to be extremely uncommon and should be expected only where the benefits of additional care are very high, or the cost is unusually low [1]. The effective female bias induced by limited territory space, which is common among polygamous cichlid species [66]–[69], may be a potent factor underlying the remarkable consistency of maternal only care exhibited by the haplochromine species in Lakes Malawi and Victoria [66]. Several recent models of cichlid speciation suggest that transient skews in the operational sex ratio may be caused when the risk of inbreeding is high during a population bottleneck [70]–[72]. These conditions would be favorable for the invasion of a dominant female determiner, resulting in a female-biased population [70]–[72]. While fluctuations in the operational sex ratio may foster the maintenance of labile parental care strategies, all of these models hypothesize that the resulting skew would be female biased. In addition, with the exception of the biparental cichlid *Eretmodus cyanostictus* [73], there is limited evidence of male-biased populations in the field.

Even if male-biased populations were more common, modeling of *Sarotherodon galilaeus* parental care behavior suggests that male bias must be coupled with large clutch sizes in order for biparental mouthbrooding to be a stabile strategy [19]. While mouthbrooding provides superior protection for the brood from predation, it also generates a massive constraint on reproductive output, since the female is only able to carry a limited number of eggs within the buccal cavity. Experimental manipulations of a pair's capacity to carry a brood suggests that biparental care is more likely when clutch size is larger than either sex can incubate alone [9], [54], [74]. If buccal capacity is a critical determinant of parental care strategy, then we would expect biparental mouthbrooding species to exhibit smaller buccal cavities, higher fecundities, and/or larger eggs, when compared to the closely related species that practice maternal only mouthbrooding. A systematic analysis to test this hypothesis is currently underway.

### Proximate basis of mating strategies

Rates of parallelism are often high in rapidly evolving clades and are commonly interpreted as evidence of natural selection [75]. Parallelism of morphological traits has been particularly well studied in sticklebacks [75], [76], cave dwelling organisms [77], and anolis lizards [78]. The extraordinary radiations of cichlid fishes in East Africa exhibit parallelism for habitat preferences [28], sexually selected traits [46], [47], opsin gene expression [79], life history traits [80], and trophic morphology between [81] and within lakes [82]. The results of our study demonstrate that evolution can also lead to rapid parallel transitions in mating and parental care strategies.

Since biparental care usually co-occurs with monogamous mating systems and maternal only care is most common in polygamous mating systems [16], the labile evolution of mating strategy within this clade provides us with a unique opportunity to examine the proximate mechanisms underlying mate choice decisions. Synchrony between the male and female is less critical for polygamous species where the males are in a constant state of reproductive readiness and where females assess and choose mates after final egg maturation. In contrast, mate choice in monogamous species occurs during the formation of the pair bond, which typically occurs a week prior to the reproductive event [16], [83]. The repeated transitions between mating strategies within the *Xenotilapia* lineage (Fig. 3) would necessitate the repeated evolution of neural and endocrine pathways leading to mate choice decisions. Since monogamous species perform mate assessment during pair bond formation, days prior to spawning [16], [83], and polygamous species perform multiple levels of mate assessment at the moment of spawning [84], [85], females that employ different mating strategies make mate choice decisions under different hormonal backgrounds [83].

There is growing evidence that rapid parallel evolution often involves the repeated recruitment of the same genes or physiological processes [86], [87]. In sticklebacks, the repeated evolution for the reduction in body armor observed in freshwater species is the result of repeated fixation of a specific haplotype of the ectodysplasin gene, which exists in low frequency in the marine species [88]. The repeated evolution of reduced pigmentation in mammals has been associated with changes in the melanocortin-1 receptor [82]. Variation in the function of the neuropeptide arginine vasopressin and its receptor have been implicated in affiliative behavior and pairbonding in a broad range of vertebrates [89]–[93]. Elucidating whether or not the same genes have been repeatedly recruited during transitions between mating/parental care strategies within the *Xenotilapia* lineage will require a careful examination of gene expression within a comparative context [94].

### Conclusions

Our analysis supports previous findings [28], [33] that the genus *Xenotilapia* is paraphyletic with respect to the genus *Enantiopus* and is in need of revision. In addition, we have identified a surprising number of parallel transitions from maternal only to biparental mouthbrooding (Fig. 3). Finally, we suggest that the incredible evolutionary lability of parental care/mating systems of the *Xenotilapia* lineage presents us with a powerful model system in which to elucidate the molecular basis and evolution of alternative mating strategies.
